# Integrated RNA-Seq and DAP-Seq Analyses Identify a DntMYB1-Centered Regulatory Module Controlling Purple Flower Formation in Nobile-Type *Dendrobium*

**DOI:** 10.3390/plants15101587

**Published:** 2026-05-21

**Authors:** Yuying Yin, Jieqiu Wu, Jie Li, Zhiyong Tan, Junqiang Fan, Huacai Zhuang, Zaowen Li, Haiping Fu, Cong Xu

**Affiliations:** 1 Dongguan Research Center of Agricultural Sciences, Dongguan 523086, China; yinyuying18@mails.ucas.edu.cn (Y.Y.); jieqiu2021520@163.com (J.W.); tanzy@163.com (Z.T.); fanjunqiang3427@163.com (J.F.); zhuanghc@163.com (H.Z.); lizaowen@126.com (Z.L.); 13825750036@139.com (H.F.); 2 Guangdong Provincial Key Laboratory of Ornamental Plant Germplasm Innovation and Utilization, Environmental Horticulture Research Institute, Guangdong Academy of Agricultural Sciences, Guangzhou 510640, China; goodlilie666@163.com

**Keywords:** nobile-type *Dendrobium*, flower color, anthocyanin, R2R3-MYB, DAP-seq, RNA-seq

## Abstract

Flower color is a key ornamental trait in Nobile-type *Dendrobium*, yet the molecular basis underlying purple flower formation remains poorly understood. In this study, a white-flowered paternal cultivar and its purple-flowered filial line were used to investigate the regulatory mechanism of purple floral pigmentation. Comparative phenotypic analysis showed that floral color divergence was established early during flower development, and anthocyanidin profiling of full-bloom petals revealed that purple flowers accumulated substantially higher levels of most anthocyanidins than white flowers, with delphinidin- and cyanidin-derived anthocyanidins together accounting for 80.26% and 94.56% of the total anthocyanidins in white- and purple-flowered materials, respectively. Transcriptome profiling identified a total of 21,235 differentially expressed genes (DEGs), with significant enrichment of phenylpropanoid- and flavonoid-related pathways, and MYB transcription factors prominently represented among the candidate regulators across the three comparison groups. Among them, *DntMYB1* was identified as a C1 subgroup R2R3-MYB associated with floral pigmentation, and transient overexpression assays in *Phalaenopsis* hybrid V3 and a Nobile-type *Dendrobium* hybrid supported its positive role in visible purple pigmentation. By integrating RNA-seq and DAP-seq analyses, we identified 3205 candidate downstream targets of DntMYB1 and established a DntMYB1-centered regulatory module. Among these candidates, one *Dnt4CL1* gene and one *DntF3′H1* gene were validated as robust direct targets of DntMYB1 through yeast one-hybrid, EMSA, and dual-luciferase assays. These findings suggest that DntMYB1 is associated with purple flower formation by coordinately regulating both upstream precursor metabolism and downstream anthocyanin biosynthesis, providing new insight into the molecular regulation of flower color in Nobile-type *Dendrobium* and useful candidate genes for ornamental trait improvement.

## 1. Introduction

Nobile-type *Dendrobium* is a horticultural group of the genus *Dendrobium* (Orchidaceae), mainly derived from *Dendrobium nobile* and its related species native to southwestern and southern China via artificial hybridization and selective breeding [1]. Unlike monopodial orchids such as *Phalaenopsis*, Nobile-type *Dendrobium* exhibits sympodial growth. After each pseudobulb completes its growth, apical development ceases and new lateral buds are initiated from the basal region, subsequently developing into new pseudobulbs [2]. Flower bud differentiation is strongly promoted by diurnal temperature fluctuation, and floral buds emerge directly from the nodes of mature pseudobulbs, usually producing one to three flowers per node [3]. With profuse blooms, lingering fragrance, and rich color variation, Nobile-type *Dendrobium* possess high ornamental value, making them a favored option for potted ornamental. In this group, pink-to-purple tones are the most striking floral coloration, often accompanied by dark purple pigmentation at the tips of the two petals and three sepals, forming the characteristic ‘quinquevulnera’ pattern. Purple thus represents a key basal tone underlying floral color diversification in Nobile-type *Dendrobium*. However, the molecular basis of floral pigmentation in this group remains poorly understood.

Floral coloration in plants is determined by different classes of pigments, mainly including chlorophylls, carotenoids, betalains, and anthocyanins. Unlike chlorophylls and carotenoids, which are localized in plastids, anthocyanins are flavonoid compounds stored in vacuoles and are primarily responsible for red, purple, and blue coloration in flowers [4,5]. Nearly 650 naturally occurring anthocyanins have been identified, among which the most common anthocyanidin backbones are delphinidin, pelargonidin, cyanidin, petunidin, malvidin, and peonidin [6]. In orchids such as *Phalaenopsis*, purple floral coloration is largely determined by anthocyanin accumulation, with cyanidin derivatives representing major contributors to purple pigmentation [7,8]. Similarly, in *Dendrobium*, the formation of purple–red floral coloration has also been associated with cyanidin-related anthocyanins [9,10]. Anthocyanin biosynthesis is one of the best-characterized secondary metabolic pathways in plants. The pathway can be roughly divided into three stages: conversion of phenylalanine to coumaroyl-CoA, formation of dihydroflavonols from coumaroyl-CoA, and the subsequent synthesis of diverse anthocyanins. In this process, *CHS* (*chalcone synthase*) plays a key role in the early flavonoid branch, whereas *F3′H* (*flavonoid 3′-hydroxylase*), *F3′5′H* (*flavonoid 3′,5′-hydroxylase*), and *DFR* (*dihydroflavonol 4-reductase*) are critical enzymes that largely determine anthocyanin composition [6]. Previous studies in *Dendrobium* have shown that structural genes in this pathway display dynamic expression patterns during flower development. Whang et al. [11] isolated *DFR*, *CHS*, and *F3′5′H* genes from *Dendrobium*. While *F3′5′H* exhibited high transcriptional levels specifically at the base of the column attached to the petals, no significant differential expression of *DFR* or *CHS* was observed across various floral organs. Following the transient transformation of white tepals via particle bombardment, *F3′5′H* was successfully expressed, whereas *DFR* and *CHS* were not. Furthermore, Sahagun et al. [12] demonstrated that *F3′H* expression in *Dendrobium* ‘Sonia Darsakul’ progressively increases during floral anthesis, slightly declining after full bloom. Notably, silencing of this gene was shown to effectively block pigment biosynthesis. These results suggest that both *F3′H* and *F3′5′H* function as pivotal structural genes in the biosynthesis of anthocyanins, determining the formation of purple coloration and distinct floral patterning in *Dendrobium*.

MYB proteins constitute one of the largest transcription factor families in plants. Based on the number of imperfect MYB repeats, MYB proteins are generally classified into four subfamilies, namely R1-MYB, R2R3-MYB, R1R2R3-MYB, and 4R-MYB. Among them, R2R3-MYB transcription factors play central roles in regulating anthocyanin biosynthesis by directly modulating the expression of structural genes in the pathway [13,14]. In monocotyledonous plants, including orchids, anthocyanin-related R2R3-MYB regulators are often assigned to the C1-like subgroup. In maize, the *C1* gene was the first *MYB* gene shown to specifically regulate anthocyanin biosynthesis in the aleurone layer [15]. In *Phalaenopsis*, *PeMYB2*, *PeMYB11*, and *PeMYB12* have been demonstrated to regulate anthocyanin accumulation in the solid red regions of sepals and petals, red spots, and venation-associated regions, respectively [16]. In *Dendrobium*, MYB-mediated regulation of anthocyanin biosynthesis has also been increasingly reported. In *Dendrobium* hybrids, *DhMYB2* cooperates with *DhbHLH1* to activate several late biosynthetic genes, including *DhF3′H1*, *DhF3′5′H2*, *DhDFR*, *DhANS*, and *DhGT4*, thereby promoting anthocyanin accumulation [17,18]. In *Dendrobium nobile*, transcriptome and metabolome analyses have identified several structural genes associated with flower color variation, such as *DnF3′H*, *DnDFR*, and *DnGT1* [19]. More recently, *DnMYB1* and *DnMYB2* were found to participate in perianth pigmentation patterning together with MADS-box factors, and *DnF3H*, *DnFLS*, and *DnBZ1* were identified as important downstream targets [20]. However, current studies on *Dendrobium* have primarily focused on candidate gene screening, MYB-bHLH complex-mediated activation of late biosynthetic genes, and the regulatory mechanisms underlying regional pigmentation patterns. Consequently, the genome-wide direct targets and hierarchical regulatory networks of MYB transcription factors involved in anthocyanin biosynthesis remain largely unknown. To address these gaps, a white-flowered paternal cultivar and its purple-flowered filial line were employed. Leveraging the highly similar genetic backgrounds of these two materials was used to minimize genomic interference. Meanwhile, this study conducted an in-depth investigation by integrating RNA-seq and DAP-seq analyses to decipher the hierarchical regulatory modules and transcriptional mechanisms governing anthocyanin biosynthesis.

Floral morphology and anthocyanin accumulation of white-flowered and purple-flowered were compared, followed by transcriptome profiling to identify candidate regulators associated with purple pigmentation. A key R2R3-MYB transcription factor, designated *DntMYB1*, was subsequently isolated and functionally characterized via transient overexpression assays. By integrating RNA-seq and DAP-seq analyses, candidate downstream targets of DntMYB1 involved in the anthocyanin biosynthetic pathway were systematically identified. Further molecular assays demonstrated that DntMYB1 directly binds to and activates a *Dnt4CL* gene (designated *Dnt4CL1*) and a *DntF3′H* gene (designated *DntF3′H1*), thereby promoting anthocyanin biosynthesis and contributing to purple flower formation in Nobile-type *Dendrobium*. Together, these findings reveal a DntMYB1-centered regulatory module underlying purple flower formation and provide a useful molecular framework for ornamental trait improvement in *Dendrobium*.

## 2. Materials and Methods

### 2.1. Plant Materials and Growing Conditions

The white-flowered material used in this study is *Dendrobium* Lucky Girl ‘Sweetheart’, and the purple-flowered material is an F_1_ progeny derived from using *Dendrobium* Lucky Girl ‘Sweetheart’ as the paternal parent. These materials were used for phenotypic observation, transcriptome sequencing, and anthocyanin quantification. *Phalaenopsis* ‘V3’ and Nobile-type *Dendrobium* ‘Sandu’ cultivar were used for transient transformation experiments. All plants were cultivated in a greenhouse at the Dongguan Research Center of Agricultural Sciences (Dongguan, China). The plants were grown under natural light conditions filtered through a 60% shade cloth, with temperatures ranging from 15 to 32 °C and relative humidity maintained between 75% and 99%.

### 2.2. Targeted Metabolomic Analysis of Anthocyanins

Petals at the full-bloom stage from white-flowered and purple-flowered *Dendrobium* were collected for anthocyanin analysis. Each group included three biological replicates (0.05 g per replicate). Anthocyanin-targeted metabolite profiling was performed using a UHPLC-MRM-MS/MS platform (Biomarker Technologies, Beijing, China). Briefly, samples were extracted with methanol–water solution (7:3, *v*/*v*) containing 0.1% formic acid, followed by centrifugation and filtration prior to analysis. Chromatographic separation was conducted using a Waters ACQUITY UPLC HSS T3 column (100 mm × 2.1 mm, 1.8 μm) (Waters, Milford, MA, USA). The mobile phase consisted of water and acetonitrile, both containing 0.1% formic acid. Mass spectrometry analysis was performed on a SCIEX QTRAP 6500+ system (SCIEX, Framingham, MA, USA) equipped with an electrospray ionization (ESI) (SCIEX, Framingham, MA, USA) source in multiple reaction monitoring (MRM) mode. Quantification was based on calibration curves generated from standard solutions. Each sample was analyzed in triplicate, and the data are presented as the mean ± standard error (SE).

### 2.3. RNA Isolation and Transcriptome Sequencing

Total RNA was extracted from petal tissues of white-flowered and purple-flowered Nobile-type *Dendrobium* plants at three developmental stages (W1/W2/W3 and P1/P2/P3). RNA extraction was performed using RNAiso Plus (TaKaRa, Dalian, China) following the manufacturer’s instructions. RNA quality was assessed using an Agilent 2100 Bioanalyzer (Agilent Technologies, Santa Clara, CA, USA) and RNA concentration was measured with a NanoDrop ND2000 spectrophotometer (Thermo Scientific, Waltham, MA, USA). A total of 18 cDNA libraries (two phenotypes × three developmental stages × three biological replicates) were constructed using the NEBNext^®^ Ultra™ RNA Library Prep Kit for Illumina^®^ (NEB, Ipswich, MA, USA) and sequenced on an Illumina NovaSeq 6000 platform (Biomarker Technologies Co., Beijing, China). Raw reads were filtered using fastp to remove low-quality reads and adapter sequences, and the remaining high-quality reads were defined as clean reads. These clean reads were mapped to the *Dendrobium nobile* reference genome (GCA_022539455.1) using HISAT2. Gene expression levels were calculated as fragments per kilobase of transcript per million mapped reads (FPKM). Differentially expressed genes (DEGs) were identified using DESeq2, with thresholds of false discovery rate (FDR) < 0.01 and fold change ≥ 2 Functional annotation of DEGs was performed using Kyoto Encyclopedia of Genes and Genomes (KEGG) enrichment analyses.

### 2.4. Identification, Expression Analysis, Phylogenetic Analysis, and Transient Expression Analysis of DntMYB1

The full-length coding sequence of *DntMYB1* was obtained from transcriptome data and amplified by PCR using gene-specific primers (Supplementary Materials, Table S1). To examine its expression pattern, total RNA was extracted from petal tissues collected at different developmental stages, and first-strand cDNA was synthesized by reverse transcription. qRT-PCR was performed using gene-specific primers, with *Dntactin* used as the internal reference gene. PCR were conducted in a LightCycler 480 II system (Roche, Mannheim, Germany) using the PerfectStart Green qPCR SuperMix (TransGen Biotech Co., Ltd., Beijing, China). Relative expression levels were calculated using the 2^−ΔΔCt^ method. Three biological replicates were analyzed for each sample. For sequence analysis, full-length amino acid sequences of MYB transcription factors were aligned using DNAMAN (v8.0). Phylogenetic analysis was conducted using MEGA (v7.0) with the neighbor-joining (NJ) method and 1000 bootstrap replicates. Reference R2R3-MYB protein sequences were retrieved from the NCBI GenBank database, and their accession numbers are listed in the Supplementary Materials, Table S2.

*DntMYB1* was inserted into the pSuper1300-GFP vector using a Trelief^®^ Seamless Cloning Kit (Tsingke, Beijing, China). The recombinant plasmid was introduced into *Agrobacterium tumefaciens* strain EHA105. Positive colonies were selected and verified by colony PCR. A single colony was cultured in LB medium containing rifampicin (25 μg/mL) and kanamycin (50 μg/mL) at 28 °C until the OD600 reached 0.8–1.0. Bacterial cells were collected and resuspended in infiltration buffer (1 mM MES, 1 mM MgCl_2_, and 100 μM acetosyringone) to a final OD600 of 0.8, followed by incubation in the dark for 2–3 h. For transient expression assays, the bacterial suspension was infiltrated into the abaxial side of petals using a needleless syringe. After infiltration, plants were incubated in the dark for 24 h and then transferred to normal growth conditions for 3–7 days. Plants infiltrated with the empty vector were used as negative controls.

### 2.5. DAP-Seq Analysis

The coding sequence (CDS) of *DntMYB1* was cloned into a protein expression vector and verified by sequencing. The recombinant protein was expressed in vitro using a cell-free expression system and confirmed by Western blot analysis. Genomic DNA was extracted from plant tissues, fragmented, and used to construct sequencing libraries. The expressed DntMYB1 protein was incubated with affinity magnetic beads and subsequently mixed with the genomic DNA library for DNA–protein binding. After washing to remove unbound DNA fragments, the specifically bound DNA was eluted and amplified to construct the final library. Sequencing was performed on an Illumina NovaSeq platform with paired-end 150 bp reads. Raw sequencing data were processed using fastp for quality control, and clean reads were aligned to the reference genome using BWA-MEM. Peak calling was performed using MACS2 with input DNA as a control. Conserved binding motifs were identified using MEME-ChIP, and peak annotation was conducted using ChIPseeker. Motif distribution bound by DntMYB1 on selected genes were shown using IGV (2.19.7).

### 2.6. Yeast One-Hybrid (Y1H) Assay

The promoter regions of *DntF3′H1* and *Dnt4CL1* were amplified and cloned into the pAbAi vector to generate bait constructs. The recombinant plasmids were linearized and transformed into Y1HGold yeast cells to generate bait strains. For interaction assays, the coding sequence of *DntMYB1* was cloned into the pGADT7 vector and co-transformed into the bait strains. Yeast cells transformed with pAbAi-53/pGADT7 were used as negative controls, while pAbAi-53/pGADT7-p53 served as positive controls. Transformed yeast cells were cultured on SD/-Ura/-Leu medium supplemented with increasing concentrations of aureobasidin A (AbA). Yeast growth under selective conditions was used to determine protein–DNA interactions.

### 2.7. Electrophoretic Mobility Shift Assay (EMSA)

The CDS of *DntMYB1* was cloned into the pMAL-c5x expression vector and transformed into *Escherichia coli* ArcticExpress (DE3) cells for protein expression. The recombinant protein was purified using Ni-affinity chromatography. DNA probes were designed based on predicted binding motifs and DAP-seq results (Supplementary Materials, Table S1). For specificity analysis, unlabeled competitor probes (cold probes) and mutant probes were included in the EMSA. Binding reactions between purified protein and labeled probes were performed, followed by separation on native polyacrylamide gels. After electrophoresis, signals were detected to assess DNA–protein interactions.

### 2.8. Dual-Luciferase (Dual-LUC) Assay

The CDS of *DntMYB1* was cloned into the pGreenII 62-SK vector as the effector construct. Promoter fragments (~2000 bp) of *DntF3′H1* and *Dnt4CL1* were inserted into the pGreenII 0800-LUC vector to generate reporter constructs, in which firefly luciferase (LUC) was used as the reporter and renilla luciferase (REN) as the internal control. The constructs were introduced into Agrobacterium tumefaciens strain EHA105. Bacterial cultures were resuspended in infiltration buffer (10 mM MES, 10 mM MgCl_2_, and 150 μM acetosyringone, pH 5.6) and adjusted to an OD600 of approximately 0.8. Effector and reporter strains were mixed at a ratio of 10:1 (*v*/*v*) and incubated at room temperature for 2–3 h. The mixtures were infiltrated into the abaxial side of leaves of 4 to 6 week-old *Nicotiana benthamiana*. After incubation in the dark for 24 h, plants were grown under normal conditions for an additional 2 days. Luciferase activities were measured using a dual-luciferase reporter assay system (Coolaber, Beijing, China). The LUC/REN ratio was calculated to represent relative promoter activity. In addition, in vivo imaging was performed after application of luciferin substrate.

## 3. Results

### 3.1. Morphological Characteristics During Flower Development and Anthocyanin Accumulation in White- and Purple-Flowered Dendrobium

**Figure 1:**
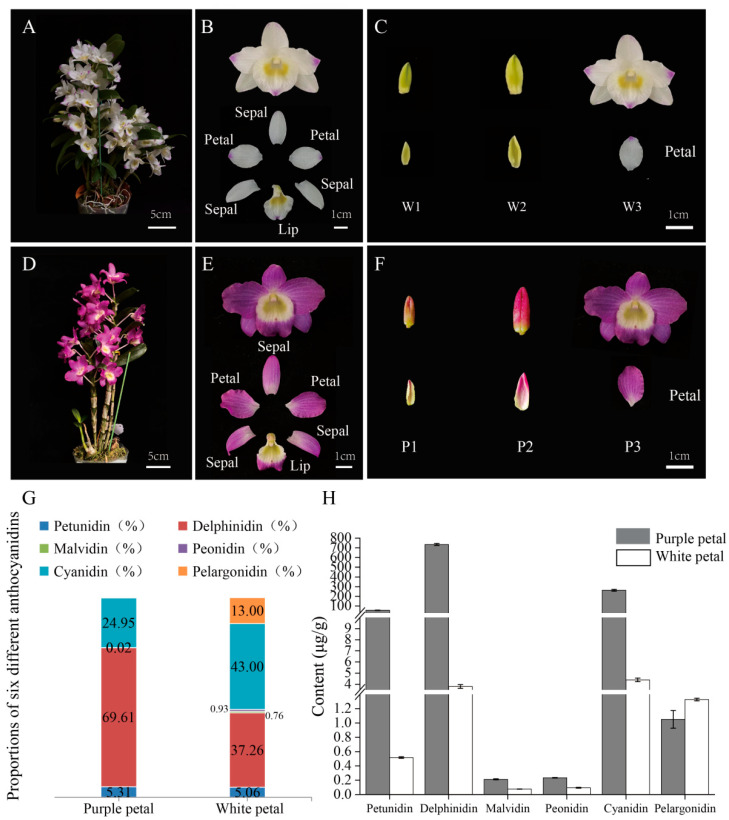
**Morphological characteristics and anthocyanin profiles of white- and purple-flowered *Dendrobium*.** (**A**,**D**) Whole-plant phenotypes of white-flowered and purple-flowered *Dendrobium*, respectively. Scale bars = 5 cm. (**B**,**E**) Floral morphology and dissected floral organs of white- and purple-flowered plants. Sepals, petals, and lip are indicated. Scale bars = 1 cm. (**C**,**F**) Flower buds and dissected petals at three developmental stages in white-flowered (W1–W3) and purple-flowered (P1–P3) plants. W1/P1: early stage (≤1 cm); W2/P2: middle stage (1–1.5 cm); W3/P3: full-bloom stage. Scale bars = 1 cm. (**G**) Proportions of six anthocyanidins in petals at stages W3 (white-flowered petals) and P3 (purple-flowered petals). (**H**) Contents of six anthocyanidins in petals at stages W3 (white-flowered petals) and P3 (purple-flowered petals) (μg/g). Error bars indicate standard deviation.

A white-flowered paternal cultivar and its purple-flowered filial line exhibited distinct differences in floral coloration throughout development (Figure 1A,D). In the white-flowered plants, flower buds at the early (≤1 cm, W1) and middle (1–1.5 cm, W2) stages were uniformly light green. The dissected inner petals at these stages also displayed a similar pale green coloration. At the full-bloom stage (W3), the flowers appeared predominantly white, with only faint purple pigmentation at the tips of the petals and the lip (Figure 1B,C). In contrast, purple-flowered plants displayed strong pigmentation throughout flower development. The buds at P1 and P2 were dark purple, and the dissected petals at these stages were mainly purple with a light green base. At the full-bloom stage (P3), the petals were almost entirely purple, and the fully opened flowers exhibited uniform purple coloration in most floral organs, except for the yellow throat region of the lip (Figure 1E,F).

To investigate the biochemical basis underlying these color differences, six major anthocyanidins were quantified in petals collected at the full-bloom stage (W3 for white petals and P3 for purple petals), when pigmentation differences were fully established. In both phenotypes, delphinidin and cyanidin were the predominant anthocyanidins, accounting for 80.26% and 94.56% of the total anthocyanidin content in white- and purple-flowered plants, respectively (Figure 1G). Except for pelargonidin, which showed comparable levels between the two phenotypes, the contents of the other five anthocyanidins were significantly higher in purple-flowered plants than in white-flowered plants (Figure 1H). These results indicate that increased accumulation of anthocyanidins, particularly delphinidin- and cyanidin-derived anthocyanidins, is closely associated with purple pigmentation in *Dendrobium* flowers.

### 3.2. Transcriptome Profiling of Petals at Three Developmental Stages in White- and Purple-Flowered Dendrobium

To explore the molecular basis underlying floral color variation, RNA-seq was performed on petal tissues from white- and purple-flowered *Dendrobium* at three developmental stages (W1/W2/W3 and P1/P2/P3), with three biological replicates per stage, resulting in a total of 18 libraries. A total of 113.93 Gb of clean data was generated, with each library yielding more than 6 Gb of clean reads. The percentage of bases with a quality score ≥ Q30 exceeded 91.90% for all samples, indicating high sequencing quality. Detailed statistics of the clean reads are provided in the Supplementary Materials, Table S3. The high-quality clean reads were mapped to the *Dendrobium nobile* reference genome, with mapping rates ranging from 78.53% to 81.80% (Supplementary Materials, Table S4). Based on genome annotation, a total of 29,043 unigenes were identified, and 3532 novel genes were discovered. Among these, 1590 (45.01%) were successfully annotated in at least one public database (Supplementary Materials, Table S5).

**Figure 2:**
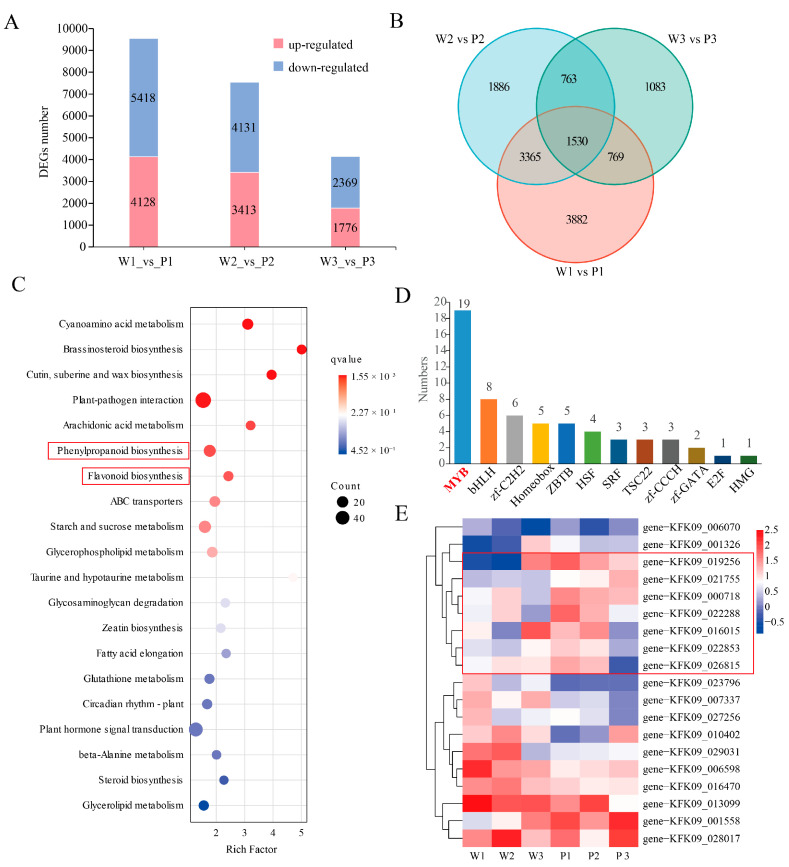
**Transcriptome analysis of white- and purple-flowered *Dendrobium* at different developmental stages.** (**A**) Number of differentially expressed genes (DEGs) identified in three comparison groups (W1 vs. P1, W2 vs. P2, and W3 vs. P3). Red and blue bars indicate up-regulated and down-regulated genes, respectively. (**B**) Venn diagram showing the overlap of DEGs among the three comparison groups. (**C**) KEGG pathway enrichment analysis of the 1530 shared DEGs. The *x*-axis represents the rich factor, the dot size indicates the number of genes, and the color scale represents the q-value. The red boxes indicate key pathways that may involved in pigment accumulation. (**D**) Distribution of transcription factor families identified from the shared DEGs. (**E**) Heatmap showing the expression patterns of selected candidate genes across different developmental stages (W1/W2/W3 and P1/P2/P3). The red box indicates seven MYB genes with higher expression in purple-flowered plants (P1, P2) than in white-flowered plants (W1, W2).

Differential expression analysis identified a total of 21,235 differentially expressed genes (DEGs) across the three comparison groups (W1 vs. P1, W2 vs. P2, and W3 vs. P3). The largest number of DEGs was detected in W1 vs. P1 (9546 genes), whereas the smallest number was observed in W3 vs. P3 (4145 genes) (Figure 2A). A total of 1530 DEGs were shared among all three comparison groups (Figure 2B). KEGG enrichment analysis of these commonly regulated DEGs revealed significant enrichment in pathways related to phenylpropanoid biosynthesis and flavonoid biosynthesis, which are closely associated with anthocyanin production (Figure 2C). To further identify potential regulatory factors, transcription factor (TF) prediction was performed on the 1530 shared DEGs. Several TF families were identified, including MYB, bHLH, and C2H2, among which the MYB family was the most abundant, comprising 19 members (Figure 2D). Expression profiling of these candidate MYB genes revealed distinct patterns between the two phenotypes across developmental stages. Notably, seven MYB genes showed consistently higher expression levels in purple-flowered plants than in white-flowered plants at the first two developmental stages (Figure 2E), suggesting that they may participate in the regulation of anthocyanin biosynthesis and purple floral pigmentation in *Dendrobium*.

### 3.3. DntMYB1 Is Involved in the Regulation of Floral Pigmentation in Dendrobium

**Figure 3:**
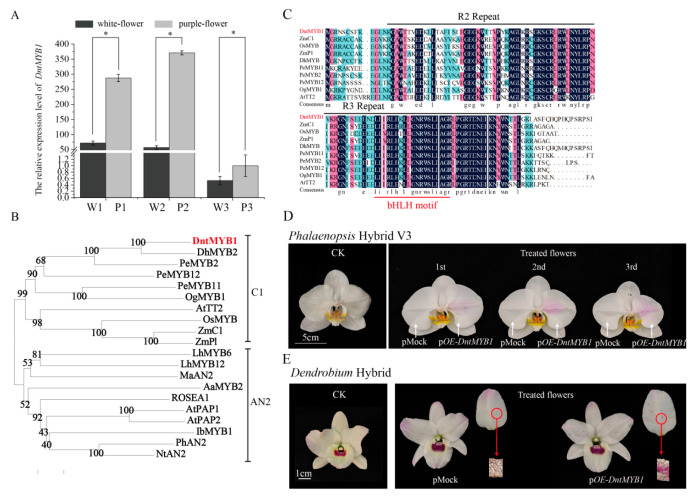
**Expression pattern, sequence characteristics, and functional analysis of *DntMYB1*.** (**A**) qRT-PCR analysis of *DntMYB1* expression in petals of white-flowered and purple-flowered *Dendrobium* at three developmental stages (S1–S3). *DntActin* was used as the internal reference gene. Data are presented as mean ± SD. Asterisks indicate significant differences between white-flowered and purple-flowered samples at the same developmental stage (* *p* < 0.05, Student’s *t*-test). (**B**) Phylogenetic analysis of DntMYB1 and representative anthocyanin-related MYB proteins from other plant species. DntMYB1 is highlighted in red. Bootstrap values are indicated at the nodes. (**C**) Multiple sequence alignment of DntMYB1 with representative R2R3-MYB proteins. Conserved R2 and R3 MYB domains are marked by black lines, and the conserved bHLH-interaction motif in the R3 domain is marked by the red line. (**D**) Transient overexpression of *DntMYB1* in petals of *Phalaenopsis* hybrid V3. Visible pink-purple pigmentation appeared in the infiltrated regions of pOE-*DntMYB1* petals, whereas no obvious pigmentation was observed in the mock control. Images were recorded 4 days after injection. Scale bar = 5 cm. (**E**) Transient overexpression of *DntMYB1* in petals of a Nobile-type *Dendrobium* ‘Sandu’. Localized purple pigmentation was observed around the injection sites in pOE-*DntMYB1* petals, whereas the mock petals remained unpigmented. Hand-cut longitudinal sections further revealed anthocyanin deposition in the pigmented regions of pOE-*DntMYB1* petals. Images were recorded 5 days after injection. Scale bar = 1 cm.

Based on transcriptome data mining, a candidate MYB transcription factor gene (gene-KFK09_022288) was isolated and designated as *DntMYB1*. qRT-PCR analysis showed that *DntMYB1* was highly expressed in petals at the early and middle developmental stages (W1/P1 and W2/P2) in both white- and purple-flowered *Dendrobium*, whereas its expression decreased markedly at the full-bloom stage (W3/P3) (Figure 3A). Notably, comparison between the two phenotypes revealed that *DntMYB1* expression was consistently and significantly higher in purple-flowered plants than in white-flowered plants at all three developmental stages, suggesting that *DntMYB1* is closely associated with purple floral pigmentation. The full-length open reading frame (ORF) of *DntMYB1* is 873 bp, encoding a protein of 291 amino acids. Phylogenetic analysis revealed that DntMYB1 clusters within the C1 subgroup of the R2R3-MYB family (Figure 3B), members of which are well known to play key roles in the regulation of anthocyanin biosynthesis [13]. Multiple sequence alignment showed that DntMYB1 contains highly conserved R2 and R3 MYB domains at the N-terminus. In addition, a conserved bHLH-interaction motif was identified in the R3 domain (Figure 3C).

To further investigate the function of *DntMYB1* in anthocyanin biosynthesis, *Agrobacterium tumefaciens* strain EHA105 carrying a *35S::DntMYB1* construct was transiently expressed in petals of *Phalaenopsis* hybrid V3 and *Dendrobium* hybrid. In *Phalaenopsis*, visible purple–red pigmentation appeared in the injected regions as early as 4 days after infiltration, whereas no obvious color change was observed in the mock control (Figure 3D). Similarly, in *Dendrobium* hybrid, localized purple–red pigmentation was observed around the injection sites at 5 days after injection (Figure 3E). Hand-cut longitudinal sections further showed clear anthocyanin deposition in the pigmented regions of petals overexpressing *DntMYB1*, whereas no comparable pigmentation was detected in the control tissues (Figure 3E). These results support that *DntMYB1* acts as a positive regulator associated with floral pigmentation in *Dendrobium*.

### 3.4. Integrated DAP-Seq and RNA-Seq Analyses Identify Anthocyanin Pathway-Related Downstream Targets of DntMYB1

**Figure 4:**
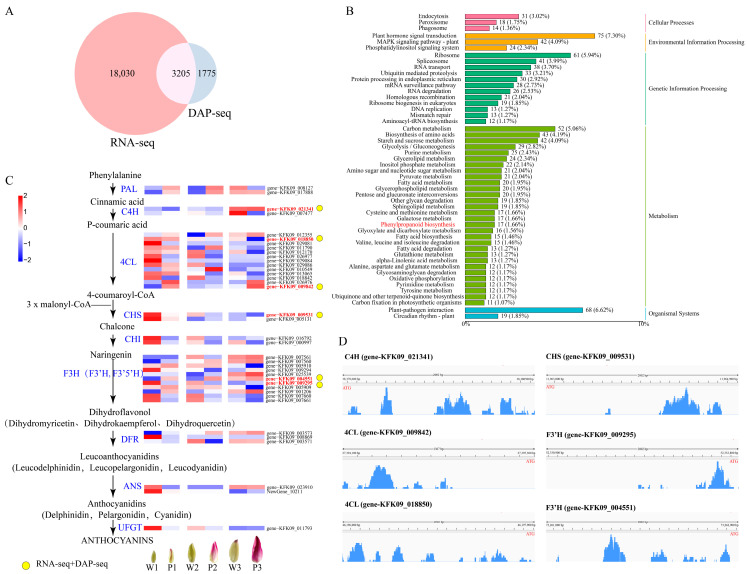
**Integrated DAP-seq and RNA-seq analyses identify anthocyanin pathway-related downstream targets of DntMYB1.** (**A**) Venn diagram showing the overlap between DntMYB1-bound genes identified by DAP-seq and differentially expressed genes (DEGs) identified by RNA-seq. A total of 3205 genes were identified as putative direct targets of DntMYB1. (**B**) KEGG classification of the 3205 target genes. Pathways related to metabolism, including phenylpropanoid biosynthesis, are highlighted. (**C**) Expression profiles of genes involved in the anthocyanin biosynthesis pathway across different developmental stages. The heatmap is based on FPKM values. (**D**) DAP-seq binding profiles of DntMYB1 on promoters of representative candidate target genes. Putative MYB-binding motifs predicted in the promoter regions are indicated, and the major DAP-seq peaks are enriched near these motifs.

To further elucidate the regulatory network of *DntMYB1* in floral pigmentation, DNA affinity purification sequencing (DAP-seq) was performed to identify its genome-wide binding targets. To determine which of these DntMYB1-bound genes were associated with floral color divergence, the DAP-seq data were integrated with the RNA-seq results. A total of 3205 genes were identified as putative direct targets that were both bound by DntMYB1 and differentially expressed between white- and purple-flowered *Dendrobium* (Figure 4A). KEGG classification analysis of these 3205 genes showed that they were mainly enriched in metabolic pathways, including carbon metabolism, starch and sucrose metabolism, and phenylpropanoid biosynthesis (Figure 4B). The significant enrichment of phenylpropanoid biosynthesis suggested that DntMYB1 may participate in floral pigmentation by directly modulating genes involved in anthocyanin biosynthesis.

To further investigate this possibility, the expression patterns of 37 genes involved in the anthocyanin biosynthesis pathway were analyzed based on their FPKM values across different developmental stages (Supplementary Materials, Table S6). A pathway-based heatmap revealed dynamic and stage-dependent expression patterns of these structural genes in white- and purple-flowered materials (Figure 4C). Some genes, including several downstream pathway genes, also showed relatively high expression at early stages in white-flowered petals, suggesting that activation of anthocyanin-related genes may occur before visible pigmentation is fully established. Among the candidate genes recovered by the integrated RNA-seq and DAP-seq analyses, one *C4H* gene (gene-KFK09_001341), two *4CL* genes (gene-KFK09_008850 and gene-KFK09_009842), one *CHS* gene (gene-KFK09_009531), and two *F3′H* genes (gene-KFK09_004551 and gene-KFK09_009295) were highlighted as potential downstream targets of DntMYB1 (Figure 4C). Notably, the two genes that were subsequently validated as direct downstream targets of DntMYB1, *4CL* (gene-KFK09_009842) and *F3′H* (gene-KFK09_004551), showed expression patterns generally consistent with purple floral pigmentation. Specifically, gene-KFK09_004551 exhibited higher expression in purple-flowered materials at all three stages, whereas gene-KFK09_009842 showed higher expression in purple flowers at stage 1 and stage 3 but no significant difference at stage 2.

Further analysis of DntMYB1 binding profiles showed that the major binding peaks associated with these candidate genes were located in their promoter regions (Figure 4D), supporting the possibility that DntMYB1 directly regulates their transcription. Together, these results suggest that DntMYB1 may control floral pigmentation by directly targeting multiple structural genes in the anthocyanin biosynthesis pathway.

### 3.5. Dnt4CL1 and DntF3′H1 Are Directly Bound and Activated by DntMYB1

To experimentally verify the candidate downstream targets identified by the integrated DAP-seq and RNA-seq analyses, promoter fragments of several anthocyanin pathway-related genes were first subjected to yeast one-hybrid (Y1H) assays. However, except for *Dnt4CL1* (gene-KFK09_009842) and *DntF3′H1* (gene-KFK09_004551), the other candidate promoters exhibited strong self-activation in yeast and were therefore unsuitable for further Y1H analysis. Consequently, these two genes were selected for subsequent validation.

**Figure 5:**
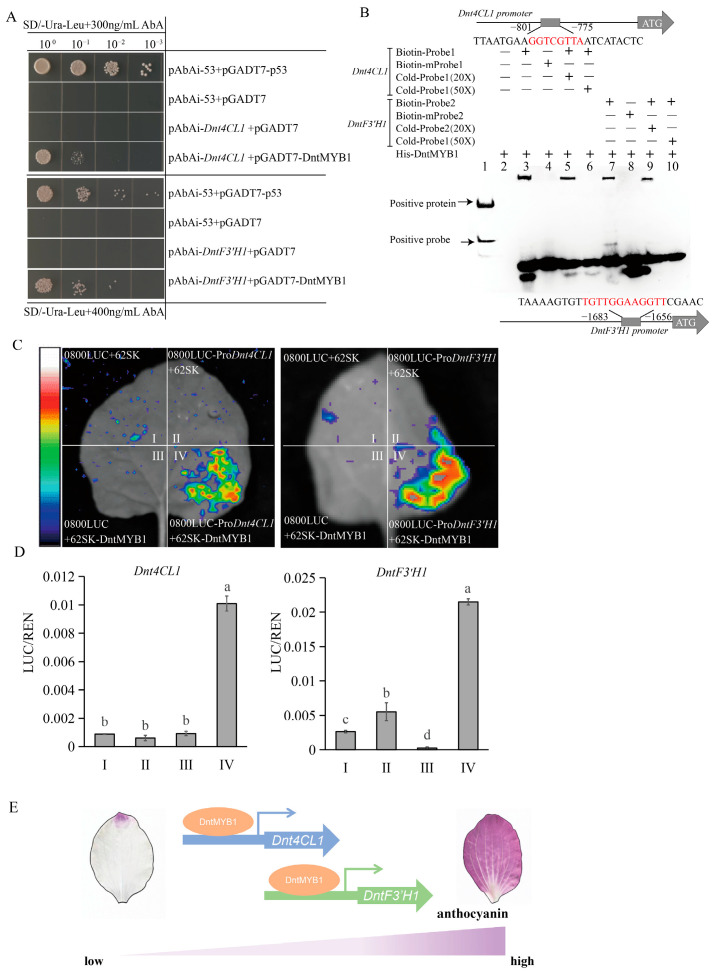
**DntMYB1 directly binds to and activates the promoters of two genes.** (**A**) Yeast one-hybrid assays showing the interaction of DntMYB1 with the promoters of one *Dnt4CL1* gene and one *DntF3′H1* gene. Yeast cells co-transformed with pGADT7-DntMYB1 and pAbAi-*Dnt4CL1* or pAbAi-*DntF3′H1* were grown on SD/-Ura/-Leu medium containing 300 ng/mL or 400 ng/mL AbA. pAbAi-53 + pGADT7-p53 was used as the positive control, while pAbAi-53 + pGADT7 and promoter reporter constructs co-transformed with empty pGADT7 served as negative controls. (**B**) Electrophoretic mobility shift assays (EMSA) showing direct binding of His-DntMYB1 to biotin-labeled promoter probes derived from the promoter regions of *Dnt4CL1* and *DntF3′H1*. Cold probe and mutant probe controls were included to verify binding specificity. The positions of the putative MYB-binding motifs in the promoter fragments are indicated above and below the gel image. (**C**) Dual-luciferase transient expression assays in *Nicotiana benthamiana* leaves showing that DntMYB1 activates the promoters of *Dnt4CL1* and *DntF3′H1*. Luminescence signals were recorded 3 days after agroinfiltration. (**D**) Quantification of dual-luciferase activity shown as the firefly luciferase to renilla luciferase ratio (LUC/REN). Different lowercase letters indicate significant differences among treatments (*p* < 0.05, one-way ANOVA). Data are presented as mean ± SD. (**E**) Proposed model for DntMYB1-mediated regulation of purple flower formation in Nobile-type *Dendrobium*. DntMYB1 directly binds to and activates the promoters of *Dnt4CL1* and *DntF3′H1*, thereby enhancing precursor metabolism and anthocyanin biosynthesis. Increased anthocyanin accumulation, mainly represented by delphinidin and cyanidin, contributes to purple floral pigmentation.

Y1H assays showed that yeast cells co-transformed with pGADT7-DntMYB1 and the promoter reporters pAbAi-*Dnt4CL1* or pAbAi-*DntF3′H1* grew normally on SD/-Ura/-Leu medium supplemented with 300 ng/mL and 400 ng/mL AbA, whereas the corresponding negative controls failed to grow (Figure 5A). These results indicate that DntMYB1 can directly associate with the promoters of both *Dnt4CL1* and *DntF3′H1* genes in yeast.

## 4. Discussion

### 4.1. Enhanced Anthocyanin Accumulation Underlies Purple Flower Formation in Nobile-Type Dendrobium

Comparative observation of white- and purple-flowered Nobile-type *Dendrobium* at different developmental stages showed that floral color divergence was established early during flower development, as purple pigmentation was already visible in the buds of the purple-flowered plants, whereas the corresponding white-flowered buds remained pale green. This developmental difference suggests that the molecular events underlying purple flower formation are initiated before anthesis, with such processes speculated to be initiated at the early stage of floral organ development. Similar developmental color divergence associated with anthocyanin accumulation has also been reported in other ornamental species, including rose [21], Petunia hybrida [22], and *Paeonia rockii* [23], where deeper floral pigmentation is generally accompanied by increased anthocyanin deposition or more spatially restricted anthocyanin accumulation.

Consistent with the visible color divergence, anthocyanidin profiling of full-bloom petals further demonstrated that purple flowers accumulated substantially higher levels of most anthocyanidins than white flowers. This result is also in agreement with previous studies in *Dendrobium*, in which purple-flowered materials accumulated higher levels of anthocyanins than white-flowered ones [18,20]. Notably, our results further showed that delphinidin and cyanidin were the predominant anthocyanidins in both white- and purple-flowered materials, although both were markedly enriched in the purple-flowered phenotype. This pattern differs somewhat from that reported in *Phalaenopsis* hybrids with red–purple, purple, purple–violet, and violet-to-violet-blue flowers, in which cyanidin-based anthocyanins accumulated to high levels whereas delphinidin was not detected [24]. By contrast, in the orchid *Glossodia major*, conspicuous floral coloration has been associated with delphinidin-based anthocyanins [25]. Taken together, these comparisons suggest that, although enhanced anthocyanin accumulation is a conserved biochemical basis of purple floral pigmentation, the relative contributions of different anthocyanidin branches may vary among orchid lineages and floral coloration types. In our Nobile-type *Dendrobium* materials, purple flower formation therefore appears to result not from the dominance of a single anthocyanidin class, but from the coordinated enhancement of both delphinidin- and cyanidin-derived pigments within a shared anthocyanidin framework.

### 4.2. DntMYB1 Functions as a Positive Regulator of Floral Pigmentation

Our expression analysis showed that *DntMYB1* was highly expressed at the bud stage in both white- and purple-flowered materials, but its transcript level declined markedly at the full-bloom stage. This temporal pattern suggests that *DntMYB1* may function primarily during the early phase of floral pigmentation. A similar expression trend has been reported for anthocyanin-related MYB regulators in *Phalaenopsis*-type *Dendrobium*, *DhMYB2* is also preferentially expressed at relatively early developmental stages in pigmented floral tissues [17,18]. Likewise, anthocyanin-related MYB genes in *Paeonia suffruticosa* ‘High Noon’ [26], *Lagerstroemia indica* [27], and rose [28] also show relatively high expression during early or intermediate flower developmental stages, followed by a decline at full bloom, suggesting that early transcriptional activation is a common feature of anthocyanin-promoting MYBs in ornamental species.

Functional assays further supported the positive regulatory role of *DntMYB1* in floral pigmentation. Transient overexpression of *DntMYB1* in *Phalaenopsis* hybrid V3 rapidly induced obvious pink-purple pigmentation in infiltrated petal regions, a phenotype similar to that reported for several anthocyanin-promoting MYB regulators isolated from orchids such as *Phalaenopsis pulcherrima* [29], *Cattleya* [30], *Cymbidium floribundum* [31] and *Cymbidium ensifolium* [32]. This indicates that the pigment-promoting function of these MYBs is at least partly conserved across orchid species. By contrast, transient overexpression of *DntMYB1* in Nobile-type *Dendrobium* ‘Sandu’ petals produced only dotted pigmentation around the injection sites, and the phenotype was much weaker than that observed in *Phalaenopsis* V3. A similar outcome was reported in the white petals of *Dendrobium* hybrid ‘Burana Charming’, in which transient expression of *DhMYB2* through a bombardment assay also produced only purple pigment spots rather than extensive pigmentation across the petal tissue [17]. The different pigmentation patterns observed between *Phalaenopsis* V3 and Nobile-type *Dendrobium* ‘Sandu’ are likely attributable to differences in the transient expression systems and host materials. Compared with *Phalaenopsis* V3, Nobile-type *Dendrobium* ‘Sandu’ petals are thinner and more papery in texture, and the flowers have a shorter display period, which may reduce the efficiency and visible extent of transient overexpression. In addition, species-specific differences in tissue sensitivity, endogenous metabolic background, and possible interference from the high polysaccharide content of *Dendrobium* may further affect *Agrobacterium*-mediated transient expression. Nevertheless, both systems consistently showed visible purple pigmentation after *DntMYB1* overexpression, supporting the conclusion that *DntMYB1* acts as a positive regulator of floral pigmentation in Nobile-type *Dendrobium*. However, an efficient loss-of-function system has not yet been established for Nobile-type *Dendrobium*, and the transient overexpression phenotype in *Dendrobium* petals was relatively localized. Therefore, future studies using gene silencing, genome editing, or other stable loss-of-function approaches will be required to further clarify the indispensability of *DntMYB1* in this process.

### 4.3. Integrated RNA-Seq and DAP-Seq Analyses Establish a DntMYB1-Centered Regulatory Module

After establishing DntMYB1 as a positive regulator of floral pigmentation, we further investigated its downstream regulatory network by integrating DAP-seq with the RNA-seq dataset generated from white- and purple-flowered Nobile-type *Dendrobium*. In plants, anthocyanin biosynthesis is commonly regulated by the conserved MYB–bHLH–WD40 (MBW) complex, in which R2R3-MYB proteins usually determine target specificity, whereas bHLH and WD40 proteins contribute to transcriptional activation and complex stabilization [33,34]. In *Arabidopsis thaliana*, anthocyanin biosynthesis is activated by MBW complexes containing PAP1/PAP2, TT8/EGL3/GL3, and TTG1 [35]. In *Petunia* hybrida, the AN2-AN1-AN11 complex is a well-established regulator of floral anthocyanin pigmentation [36], indicating that MBW-mediated regulation is a conserved mechanism underlying anthocyanin biosynthesis across plant species. In *Phalaenopsis*-type *Dendrobium*, a similar regulatory framework has also been reported DhMYB2 cooperates with DhbHLH1 to regulate anthocyanin-related genes, mainly late biosynthetic genes associated with floral pigmentation [18,20]. In our study, DntMYB1 was assigned to the C1 subgroup of R2R3-MYB proteins and contains a conserved bHLH-interaction motif, suggesting that it may also function within an MBW-like regulatory framework. However, because no direct interaction assays with bHLH or WD40 proteins were performed here, this possibility remains to be experimentally verified.

In previous studies of *Dendrobium*, the downstream targets of anthocyanin-related MYB regulators were mainly inferred from transcriptome-based candidate screening or were focused largely on late biosynthetic genes [18,20]. By contrast, our integrated analysis recovered not only the repeatedly reported anthocyanin-related target class represented by *F3′H*, but also several upstream phenylpropanoid/flavonoid pathway genes, including *C4H*, *4CL*, and *CHS.* This result suggests that DntMYB1 may potentially regulate multiple steps of the anthocyanin biosynthetic pathway rather than acting exclusively on a single late biosynthetic branch.

Importantly, although several pathway-related genes were identified as candidate downstream targets, only one *Dnt4CL* gene and one *DntF3′H* gene were supported by a complete set of molecular evidence, including Y1H, EMSA, and dual-luciferase assays. Most of the other candidate promoters showed strong self-activation in yeast, which prevented further reliable Y1H verification. Similarly, in rose, RcMYB1 was shown to broadly bind to and activate the promoters of both early biosynthetic genes (EBGs), including *RcCHSa*, *RcCHSc*, *RcCHI*, *RcF3H*, and *RcF3′H*, and late biosynthetic genes (LBGs), including *RcDFR*, *RcANS*, *RcUFGT*, and *RcGT1*, in the anthocyanin pathway [28]. In addition, another study demonstrated that the R2R3-MYB transcription factor GhMYB308 in cotton can directly bind to and activate *4CL2*, *C4H2*, and *PAL* [37]. In our study, the simultaneous regulation of *Dnt4CL1* and *DntF3′H1* by DntMYB1 is biologically meaningful because these two genes function at different levels of the pathway. *Dnt4CL1* acts at an early upstream step of the phenylpropanoid pathway and is involved in precursor supply, whereas *DntF3′H1* functions in the downstream flavonoid/anthocyanin pathway. The simultaneous regulation of these two genes by DntMYB1 ensures coordinated activation of the anthocyanin biosynthetic pathway. This regulatory architecture prevents metabolic bottlenecks, balances precursor supply with mid-to-downstream demand, and thereby enables stable purple pigmentation during flower development. Thus, DntMYB1 orchestrates both upstream precursor metabolism and mid-to-downstream anthocyanin biosynthesis, rather than targeting only a single late biosynthetic gene. From an evolutionary perspective, such a regulator controlling both the entry and exit points of a metabolic pathway represents a highly efficient strategy for regulating secondary metabolism in plants. Taken together, our results establish a DntMYB1-centered regulatory module linking transcriptional regulation to both upstream precursor metabolism and downstream anthocyanin biosynthesis.

## 5. Conclusions

In this study, parental and progeny individuals were used to investigate the molecular basis underlying purple flower formation in Nobile-type *Dendrobium*. Purple floral pigmentation was associated with enhanced anthocyanin accumulation, with delphinidin and cyanidin representing the predominant anthocyanidins in both phenotypes. Transcriptome analysis identified *DntMYB1* as a candidate regulator associated with purple pigmentation. Transient overexpression assays supported its positive role in floral coloration. To further investigate how *DntMYB1* may influence anthocyanin biosynthesis, integrated RNA-seq and DAP-seq analyses were conducted to identify potentially downstream structural genes. Multiple candidate downstream genes involved in the anthocyanin pathway were identified. Among these candidate genes, *Dnt4CL1* and *DntF3′H1* genes were validated as robust direct targets of DntMYB1 through Y1H, EMSA, and dual-luciferase assays. These findings suggest that DntMYB1 may contribute to purple flower formation by coordinately regulating both upstream precursor metabolism and downstream anthocyanin biosynthesis. Notably, integrated DAP-seq and RNA-seq analyses enabled more precise identification of potential downstream target genes than RNA-seq alone. Overall, this study expands our understanding of the transcriptional regulation of flower color in Nobile-type *Dendrobium* and provides useful candidate genes for ornamental trait improvement.

## Data Availability

The raw sequencing reads have been submitted to the NCBI database under BioProject accession PRJNA1456543.

## Supplementary Materials

The following supporting information can be downloaded at: https://www.mdpi.com/article/10.3390/plants15101587/s1. Table S1. Primers used in this study; Table S2.: Accession numbers of R2R3-MYB proteins used for phylogenetic and sequence alignment analyse; Table S3. A summary of the sequencing data; Table S4. Statistics on data mapping; Table S5. Annotated new genes statistics of different databases; Table S6. Genes involed in the anthocyanin biosynthesis.

## References

[1] Ketsa S., Warrington I.J. (2023). The *Dendrobium* orchid: Botany, horticulture, and utilization. Crop Sci..

[2] Jain A., Sarsaiya S., Gong Q., Wu Q., Shi J. (2024). Pharmacological and therapeutic biofunction of *Dendrobium nobile*: A critical review. Nat. Prod. Commun..

[3] Yen C.Y.-T., Starman T.W., Wang Y.T., Niu G. (2008). Effects of cooling temperature and duration on flowering of the nobile *Dendrobium* orchid. HortScience.

[4] Bueno J.M., Sáez-Plaza P., Ramos-Escudero F., Jiménez A.M., Fett R., Asuero A.G. (2012). Analysis and antioxidant capacity of anthocyanin pigments. Part II: Chemical structure, color, and intake of anthocyanins. Crit. Rev. Anal. Chem..

[5] Kellenberger R.T., Glover B.J. (2023). The evolution of flower colour. Curr. Biol..

[6] Lu Z., Wang X., Lin X., Mostafa S., Zou H., Wang L., Jin B. (2024). Plant anthocyanins: Classification, biosynthesis, regulation, bioactivity, and health benefits. Plant Physiol. Biochem..

[7] Hsu C.C., Su C.J., Jeng M.F., Chen W.H., Chen H.H. (2019). A HORT1 Retrotransposon Insertion in the *PeMYB11* Promoter Causes Harlequin/Black Flowers in *Phalaenopsis* Orchids. Plant Physiol..

[8] Meng X., Li G., Gu L., Sun Y., Li Z., Liu J., Wu X., Dong T., Zhu M. (2020). Comparative metabolomic and transcriptome analysis reveal distinct flavonoid biosynthesis regulation between petals of white and purple *Phalaenopsis amabilis*. J. Plant Growth Regul..

[9] Saito N., Toki K., Uesato K., Shigihara A., Honda T. (1994). An acylated cyanidin glycoside from the red-purple flowers of *Dendrobium*. Phytochemistry.

[10] Jia N., Wang J.J., Liu J., Jiang J., Sun J., Yan P., Sun Y., Wan P., Ye W., Fan B. (2021). *DcTT8*, a bHLH transcription factor, regulates anthocyanin biosynthesis in *Dendrobium candidum*. Plant Physiol. Biochem..

[11] Whang S.S., Um W.S., Song I.J., Lim P.O., Choi K., Park K.W., Kang K.W., Choi M.S., Koo J.C. (2011). Molecular analysis of anthocyanin biosynthetic genes and control of flower coloration by flavonoid 3′, 5′-hydroxylase (*F3′ 5′ H*) in *Dendrobium moniliforme*. J. Plant Biol..

[12] Sahagun J., Ratanasut K. (2016). Development of Flavanone-3-hydroxylase (*F3′ H*) Gene Silencing System in *Dendrobium* Sonia Earsakul Flowers for Engineering Aurone Biosynthetic Pathway. Proceedings of the International Biochemistry & Molecular Biology Conference, Songkla, Thailand, 26–27 May 2016.

[13] Dubos C., Stracke R., Grotewold E., Weisshaar B., Martin C., Lepiniec L. (2010). MYB transcription factors in *Arabidopsis*. Trends Plant Sci..

[14] Yan H., Pei X., Zhang H., Li X., Zhang X., Zhao M., Chiang V.L., Sederoff R.R., Zhao X. (2021). MYB-mediated regulation of anthocyanin biosynthesis. Int. J. Mol. Sci..

[15] Cone K.C., Burr F.A., Burr B. (1986). Molecular analysis of the maize anthocyanin regulatory locus C1. Proc. Natl. Acad. Sci. USA.

[16] Hsu C.C., Chen Y.Y., Tsai W.C., Chen W.H., Chen H.H. (2015). Three R2R3-MYB transcription factors regulate distinct floral pigmentation patterning in *Phalaenopsis* spp.. Plant Physiol..

[17] Li C., Qiu J., Ding L., Huang M., Huang S., Yang G., Yin J. (2017). Anthocyanin biosynthesis regulation of *DhMYB2* and *DhbHLH1* in *Dendrobium* hybrids petals. Plant Physiol. Biochem..

[18] Wang Y., Yin H., Long Z., Zhu W., Yin J., Song X., Li C. (2022). *DhMYB2* and *DhbHLH1* regulates anthocyanin accumulation via activation of late biosynthesis genes in *Phalaenopsis*-type *Dendrobium*. Front. Plant Sci..

[19] Qiu Y., Cai C., Mo X., Zhao X., Wu L., Liu F., Li R., Liu C., Chen J., Tian M. (2023). Transcriptome and metabolome analysis reveals the effect of flavonoids on flower color variation in *Dendrobium nobile* Lindl. *Front*. Plant Sci..

[20] Wang Q., Wei X.-R., Yuan M., Hou T.Z., Liu Z.J., Zheng B.Q., Wang Y. (2026). Synergism and antagonism: Unraveling the regulatory network of perianth coloration in *Dendrobium nobile* Lindl. Plant Physiol. Biochem..

[21] Sun W., Ji N., Yang J., Zhao S., Kuwantai A., Wang L., Wang L., Feng H. (2026). The RcMYB308-*RcEGL1* module regulates cyanidin accumulation by targeting the *RcF3′H* promoter in pink roses. BMC Plant Biol..

[22] Fu Z., Jiang H., Chao Y., Dong X., Yuan X., Wang L., Zhang J., Xu M., Wang H., Li Y. (2021). Three paralogous R2R3-MYB genes contribute to delphinidin-related anthocyanins synthesis in *Petunia hybrida*. J. Plant Growth Regul..

[23] Qi F.T., Han J.N., Cheng F.Y., Zhong Y., Zhang L., Zhao Y.F., Liu X.F. (2025). The PrFRS2-PrMYB75a module regulates petal coloration in flare tree peony (*Paeonia rockii*). Hortic. Res..

[24] Liang C.Y., Rengasamy K.P., Huang L.M., Hsu C.C., Jeng M.F., Chen W.H., Chen H.H. (2020). Assessment of violet-blue color formation in *Phalaenopsis* orchids. BMC Plant Biol..

[25] Wong D.C.J., Wang Z., Perkins J., Jin X., Marsh G.E., John E.G., Peakall R. (2024). The road less taken: Dihydroflavonol 4-reductase inactivation and delphinidin anthocyanin loss underpins a natural intraspecific flower colour variation. Mol. Ecol..

[26] Luan Y., Tao J., Zhao D. (2024). Synergistic actions of 3 MYB transcription factors underpin blotch formation in tree peony. Plant Physiol..

[27] Ni L., Wang J., Zhou F., Chen Z. (2025). Integrated multi-omics reveals Li-miR828z-LiMYB114 regulatory module controlling anthocyanin biosynthesis during flower color development in *Lagerstroemia indica*. Ind. Crops Prod..

[28] He G., Zhang R., Jiang S., Wang H., Ming F. (2023). The MYB transcription factor RcMYB1 plays a central role in rose anthocyanin biosynthesis. Hortic. Res..

[29] Wen J., Li J., Wu K., Zeng J., Li L., Fang L., Zeng S. (2025). Transcriptome analysis reveals *PpMYB1* and *PpbHLH1* promote anthocyanin accumulation in *Phalaenopsis pulcherrima* Flowers. Biomolecules.

[30] Li B.J., Zheng B.Q., Wang J.Y., Tsai W.C., Lu H.C., Zou L.H., Wan X., Zhang D.Y., Qiao H.J., Liu Z.J. (2020). New insight into the molecular mechanism of colour differentiation among floral segments in orchids. Commun. Biol..

[31] Ma S., Wang M., Li P., Guo L., Xiong L., Tian Y., Li J., Lan S., Liu Z., Ai Y. (2024). Transcriptome and metabolome analysis reveal the lip color variation in *Cymbidium floribundum*. Ornam. Plant Res..

[32] Wang M., Huang T., Peng Z., Wang S., Wu G., Xiong L., Lan S., Peng D., Liu Z.-J., Ai Y. (2025). An R2R3-MYB transcription factor regulates anthocyanin accumulation in response to temperature signals in *Cymbidium ensifolium*. Plant Physiol. Biochem..

[33] Lloyd A., Brockman A., Aguirre L., Campbell A., Bean A., Cantero A., Gonzalez A. (2017). Advances in the MYB-bHLH-WD Repeat (MBW) pigment regulatory model: Addition of a WRKY factor and co-option of an anthocyanin MYB for betalain regulation. Plant Cell Physiol..

[34] Petroni K., Tonelli C. (2011). Recent advances on the regulation of anthocyanin synthesis in reproductive organs. Plant Sci..

[35] Gonzalez A., Zhao M., Leavitt J.M., Lloyd A.M. (2008). Regulation of the anthocyanin biosynthetic pathway by the TTG1/bHLH/MYB transcriptional complex in *Arabidopsis* seedlings. Plant J..

[36] de Vetten N., Quattrocchio F., Mol J., Koes R. (1997). The *an11* locus controlling flower pigmentation in petunia encodes a novel WD-repeat protein conserved in yeast, plants, and animals. Genes Dev..

[37] Nyamaharo K.C., Huang Y., Yang Q., Zheng H., Vitalis N.E., Wang D., Guo W., Ke L., Yu D., Sun Y. (2025). The R2R3 MYB *GhMYB308* is a key regulator in lignin biosynthesis and modulates cotton plant architecture and fiber. Ind. Crops Prod..

